# Obstructive sleep apnoea triggering autonomic dysreflexia: Isn't it time for sleep studies to be routine in SCI?

**DOI:** 10.1016/j.bas.2023.102725

**Published:** 2023-12-12

**Authors:** Ameerah Alsaqobi

**Affiliations:** Department of Physical Medicine and Rehabilitation, Physical Medicine and Rehabilitation Hospital, Andalous, Kuwait

**Keywords:** Apnea, OSA, Sleep, Complication, SCI

## Abstract

Autonomic dysreflexia is a known complication after spinal cord injury. It is defined as a reflexive increase in blood pressure as a result of a painful or irritating stimulus below the level of the lesion. Although commonly caused by stimuli from the bladder, other rare triggers are still being described. Herein, we present a case with autonomic dysreflexia triggered by obstructive sleep apnoea. A 43-year-old smoker suffered a spinal cord injury after a road traffic accident secondary to multiple cervical spine vertebrae fractures. He presented to our rehabilitation centre 6 months post-injury as a case of complete spinal cord injury with C4 neurological level. Autonomic dysfunction in the form of autonomic dysreflexia was occurring on a daily basis. After exclusion of known inciting factors, it was noticed that early morning episodes of autonomic dysreflexia were persistent. He was also reporting daytime fatigue and sleepiness and multiple night-time awakenings. Therefore, work up for sleep apnoea was done. A diagnosis of moderate obstructive sleep apnoea was made. With continuous positive airway pressure treatment, his daily early morning episodes of autonomic dysreflexia settled. This case illustrates the importance of the pattern of blood pressure elevation to pinpoint specific triggers of autonomic dysreflexia.

## Highlights

• Sleep apnoea in SCI remains underdiagnosed and undertreated.
• Previously unrecognized triggers of autonomic dysreflexia are still being described in the literature.
• Sleep apnea may trigger autonomic dysreflexia.

## 1 Introduction

Autonomic dysreflexia (AD) is a well-known complication in patients with spinal cord injury (SCI). It is generally defined as a condition in which there is a reflexive increase in blood pressure in a patient with a SCI triggered by a noxious stimulus, the origin of which in the overwhelming majority of cases is the bladder. Patients usually present with flushing and sweating above the level of the lesion. Some may complain of a headache, blurry vision or chest discomfort. In some instances, it may be silent. In addition to the high blood pressure, the associated heart rate may be low, high or even normal (Krassioukov et al., 2021).

It mostly occurs in patients with an injury above the T6 level. Patients with complete and higher-level injuries are at an increased risk for AD. A wide range of triggers have been associated with AD; however, irritation from either the bladder or bowel remain the most common. Reports of rare or previously unknown triggers are still being published. It is vital to realise that SCI remains relatively understudied in the literature. Patients with no obvious triggers should have work up for possible new associations as appropriate from the history and physical examination findings. Herein, we describe a case with AD triggered by sleep apnoea.

## 2 Case presentation

We present a previously healthy 43-year-old male smoker, who was involved in a motor vehicle accident and transferred to the Emergency Department. He was diagnosed with cervical vertebrae two to seven (C2-7) posterior element fractures, severe burst fracture of cervical level six (C6) with spinal cord traction and sagittal fracture of C7 body as well as compression fracture of the second thoracic vertebra. He required anterior corpectomy of C6 with fixation and fusion of C5-7.

The patient presented to our rehabilitation centre six months after the injury. He was admitted as a case of C4 AIS-A (American spinal cord injury Association (ASIA) Impairment Scale, A (motor and sensory complete injury) with no concomitant traumatic brain injury. He had a Body Max Index (BMI) of 26. The patient was tetraplegic and totally dependent for all activities of daily living. Breathing was spontaneous without the need for a tracheostomy. He had a suprapubic catheter (SPC) insertion for his neurogenic bladder and was on bowel protocol for his neurogenic bowel. During his admission to our rehabilitation center, he developed multiple daily bouts of AD. Most episodes were associated with sweating and flushing. Occasionally, he would complain of a headache, chest discomfort, blurry vision, and nasal congestion.

All potential triggers that may have contributed to his AD specific to his case were investigated including a head to toe skin check revealed which no pressure sores. There was no chest infection, no bowel impaction, no urinary tract infection, no kinked suprapubic catheter, no sources of pain, no skin infections (intertigo, sweat rash), or constrictive clothing.

Work up for less common causes of AD including a non-contrast CT abdomen and pelvis was done it showed no evidence of urinary tract stones or appendicitis. His bowel was not distended. No other intraabdominal pathology was evident. MRI of whole spine to rule out syringomyelia was also performed.

His work up remained negative; however, it was noticed that the pattern of blood pressure elevation in the persisting episodes of AD were occurring daily in the early morning hours. He would wake up flushed and sweating with a blood pressure around 160/100. These episodes would settle spontaneously. His SPC was draining, and bladder scan would demonstrate no residual volume.

Further history revealed that he had excessive daytime fatigue, and sleepiness. Moreover, according to his caregiver, his sleep was interrupted with multiple night-time awakenings. Therefore, a level III sleep study was done and was diagnostic for moderate obstructive sleep apnoea (OSA). His apnoea hypopnea index was 24.9. The patient was kept on daily night-time continuous positive airway pressure administered via facemask, and his early morning AD episodes resolved.

## 3 Discussion

We present a case of AD triggered by OSA. After literature review, to our knowledge this is the first report of AD caused by OSA in SCI. The diagnosis of OSA in SCI may be challenging as some symptoms of OSA such as, daytime fatigue, overlap with medication side effects commonly used in this patient population. In addition, some common comorbidities such as depression in SCI may also cause symptoms that are shared with the symptomatology of OSA. We postulate that the cause of autonomic dysfunction is due to the autonomic alterations described previously in the literature in patients with OSA. Those with sleep apnoea have increased sympathetic activation during apnoeic episodes, which is shared with AD. It is plausible that this sympathetic excitation, which remains unchecked due to lack of the corrective inhibition severed by the SCI eventually progresses to AD (Clifford, 1998; Bycroft et al., 2005). However, literature is still lacking studies evaluating sleep apnoea in SCI and the information we have is mainly from studies in the general population. Nonetheless, factors that increase the risk of OSA likely apply. The likely association of OSA with AD in of itself increases the importance of early diagnosis and treatment of sleep apnoea in SCI, which as of now does not have any formal guidelines or screening recommendations even though prevalence was found to be high in the SCI population ranging from 27% up to 82% (Sankari et al., 2019). Sleep apnoea in SCI remains underdiagnosed and undertreated (Sankari et al., 2019).

Although there were no studies on the association of AD with OSA, OSA is prevalent in tetraplegics and in a study evaluating ambulatory blood pressure pattern in SCI, tetraplegics were found to have higher blood pressure at night than in the daytime and nocturnal hypertension. Whether this was related, in part, to AD or OSA was not studied. There was no information regarding signs of AD in those patients and no sleep studies were performed (Sankari et al., 2019; Goh et al., 2015).

As neither AD nor sleep apnoea occur in all patients with SCI, we don't have a reason to believe that all of those with SCI and sleep apnoea will develop AD as a consequence. Additionally, we postulate time since injury likely affects our detection of such problem. AD usually develops in the subacute and chronic stages after SCI. Our patient was admitted six months post injury. Those who are admitted to rehabilitation earlier, which is the norm, are at risk of going undetected.

Given the limitation of this study design, further research to prove the association between sleep apnoea and AD in SCI is required. Several challenges exist in studying such entity since the diagnosis of AD is based on clinical signs and symptoms as well as blood pressure measurements. Moreover, sleep apnoea per say may cause elevation in blood pressure so reliance on blood pressure measurement alone is likely to be misleading. As the treatment of sleep apnoea is well established and known to reduce mortality and morbidity, withholding such treatment is likely unethical, complicating future study designs (Pépin et al., 2022). We recognize the challenges but are hopeful that an association is observed in forthcoming studies.

## 4 Conclusion

In conclusion, further research is required to study the association between AD and OSA in SCI patients. In addition, a high index of clinical suspicion and timing of blood pressure elevation in patients with SCI is helpful in identifying certain triggers of AD.

## Declaration of competing interests

The authors declare that they have no known competing financial interests or personal relationships that could have appeared to influence the work reported in this paper.
